# Narrative Review of Complications Following DDH Treatment

**DOI:** 10.1007/s43465-021-00550-y

**Published:** 2021-10-23

**Authors:** Raghav Badrinath, Caitlin Orner, James D. Bomar, Vidyadhar V. Upasani

**Affiliations:** Orthopedics and Scoliosis, Rady Children’s Hospital San Diego, 3020 Children’s Way, MC 5062, San Diego, CA 92123 USA

**Keywords:** Developmental dysplasia of the hip, DDH less than 4 years old, Complications

## Abstract

**Background:**

The purpose of this narrative review was to survey the literature for common complications following treatment of DDH in children less than 4 years old.

**Methods:**

The Pubmed database was queried. Search result titles were reviewed to identify papers that were pertinent to the topic. Abstracts for these papers were obtained and read, and a subset of these were selected for review of the complete manuscript.

**Results:**

92 manuscripts were reviewed. Residual dysplasia, redislocation, and osteonecrosis are the primary complications of treatment in this age group. In the long term, hips without complications related to DDH treatment tend to do well, although a significant percentage of them will inevitably require joint replacement surgery.

**Conclusion:**

Although there is excellent potential for a good outcome when DDH is diagnosed and treated under age 4 years, osteonecrosis continues to be a concern with all treatment methods. A subset of patients from this young cohort will continue to have residual dysplasia or recurrent dislocation requiring return to the operating room.

## Background

Developmental dysplasia of the hip (DDH) is the most common congenital disorder in newborns, with an incidence of 2–6 per 1000 [1–3]. DDH covers a spectrum from acetabular undercoverage, to femoral head subluxation, to frank dislocation. Treatment for hip dysplasia varies by age at presentation, but all seek to achieve the same goals—to maintain the hip concentrically reduced within the acetabulum, enabling the forces between the femoral head and the acetabulum to remodel leading to resolution of the acetabular undercoverage. Residual dysplasia at maturity results in increased loading across the joint and development of early osteoarthritis, as described by Wiberg [4]. Studies demonstrate that the ability of the acetabulum to remodel diminishes after the age of 4, making early diagnosis and treatment crucial to an optimal outcome [5, 6]. After around this age, concentric reduction and acetabular coverage is best obtained with the help of pelvic and/or femoral sided osteotomies.

Several treatment algorithms have been proposed for DDH. While a variety of braces have been developed and are currently used, the Pavlik harness is the most common treatment for infants under the age of 6 months [7]. Various studies have demonstrated that the ability of the harness to keep the hip reduced decreases after the infant reaches sitting age, with increased rates of Pavlik harness failure and osteonecrosis with brace treatment beyond this age [8–10]. Beyond 6 months of age, treatment typically requires closed or open reduction and spica casting. Past 18 months of age, treatment is difficult to obtain closed and open reduction is often required, with concomitant pelvic osteotomy to improve acetabular dysplasia and keep the hip in a reduced position [11].

Osteonecrosis (ON) remains the most common complications of DDH treatment, with varying rates reported in the literature. Weinstein et al. recommend using the term proximal femoral growth disturbance (PFGD) to describe the radiographic changes observed in these patients as histologic studies have not confirmed the presence of ON or avascular necrosis (AVN) [12]. Nevertheless, the terms PFGD, ON, and AVN have been used interchangeable throughout the literature. Several studies have demonstrated that the rate of osteonecrosis depends on numerous factors, including age of the patient at presentation, the type of treatment, and severity of dysplasia. With advances in treatment and awareness of the problem, rates of ON have improved from the 40% with the Lorenz method of closed reduction in the early 1900s, to approximately 1–2% clinically significant disease today [13, 14].

The purpose of this narrative review was to survey the literature for common complications following treatment of DDH in children less than 4 years old. We include complications following Pavlik harness use, closed and open reductions, as well as single stage open reduction and osteotomies.

## Methods

**Fig. 1:**
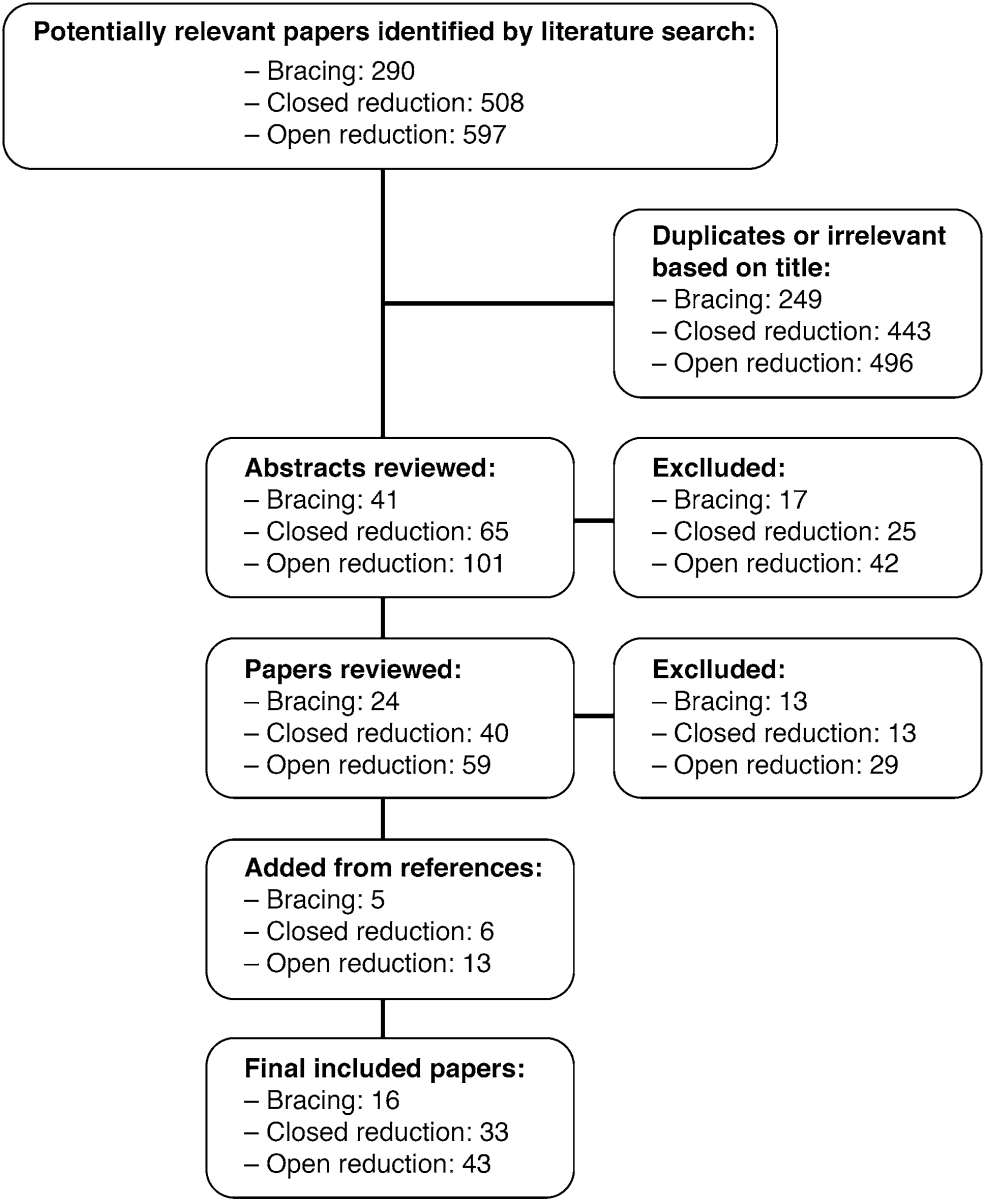
Flow diagram indicating manuscript selection

The Pubmed database was queried with MeSH search terms as documented in Table 1. For each search, paper titles were reviewed to identify papers that were pertinent to the topic. Abstracts for these papers were obtained and read, and a subset of these were selected for review of the complete manuscript. The references to these papers were also scanned to identify additional relevant papers that were not detected on initial Pubmed search. The search was limited to the English language literature from 1960 onward. Papers were excluded if they had fewer than 15 patients or had follow up of less than 6 months. Additionally, studies were excluded if outcomes or complications were not defined by well described criteria, such as the Severin grade for radiographic outcomes, or the Kalamchi and McEwen criteria for AVN. Figure 1 describes the search algorithm and step-wise progression of article selection. We specifically attempted to highlight higher level studies, or studies with larger patient populations and longer term follow up.

**Table 1:** MeSH search terms used in PubMed database

Subtopic	Search terms	# of Initial Results
Brace treatment	“Hip + dysplasia + Pavlik + complications”	79
	“Hip + dysplasia + brace + complications”	129
	“Hip + dysplasia + harness + complications”	82
Closed reduction	“Closed + reduction + hip + dysplasia + complications”	508
Open reduction	“Open + reduction + hip + dysplasia + complications”	597

## Complications Following Pavlik Harness Treatment

Early treatment for DDH involved the use of rigid braces and casts following aggressive manipulation and/or closed reduction techniques. For example, Adolf Lorenz’s technique involved traction and abduction of the hip under anesthesia, “screwing in” of the hip to displace the pulvinar and center the femoral head, followed by prolonged spica casting in abduction [15]. Although much touted for its success in reducing chronically dislocated hips, even in older children, physicians soon started looking for alternatives given a 45–50% rate of osteonecrosis. Arnold Pavlik attributed this to the violence of the reduction and maintenance of the hip in a rigid splint, calling it a “passive-mechanical” method of treatment. The idea behind the Pavlik harness was to allow for gentle reduction and active motion of the hip, theorizing that allowing motion of the joint prevents the development of constant pressure against the femoral head and thus the development of osteonecrosis [16–18]. Pavlik reported his results on 1912 hips treated in his clinic. Of these, 632 were dislocated, while 640 were either dysplastic or subluxated. Of the 632 dislocated hips, 84% reduced spontaneously with the harness with no cases of osteonecrosis observed. The 16% that failed harness treatment underwent subsequent closed reduction and bracing, with an 18% rate of ON. The overall rate of ON in the entire study population, however, was less than 1%, leading to widespread adoption of this new “functional method” of treatment [18]. We found 16 studies to be included in this review [8–10, 19–31].

Two large, multi-center studies have reported outcomes following Pavlik Harness treatment. The first, by Grill et al., report on 3611 hips in 2636 patients in a multicenter study from a European Pediatric Orthopedic Society study group [30]. Hips were followed for 1–9 years after treatment, with an average 4.46 years follow up. Patients were on average treated at age 4.1 months, and treated in the brace for an average of 6.3 months. Overall, they found the Pavlik harness was successful in reducing 92% of hips with an overall ON rate of 2.4%. As expected, higher rates of failure were correlated with older children and with severity of DDH. They found the rate of ON in children between the ages of 3 and 6 months was almost twice that of children below the age of 3 months. Similarly, they found an ON rate of 16.4% in the Tonnis grade 4 hips compared to 1.3% in the Tonnis grade 1 hips.

**Fig. 2:**
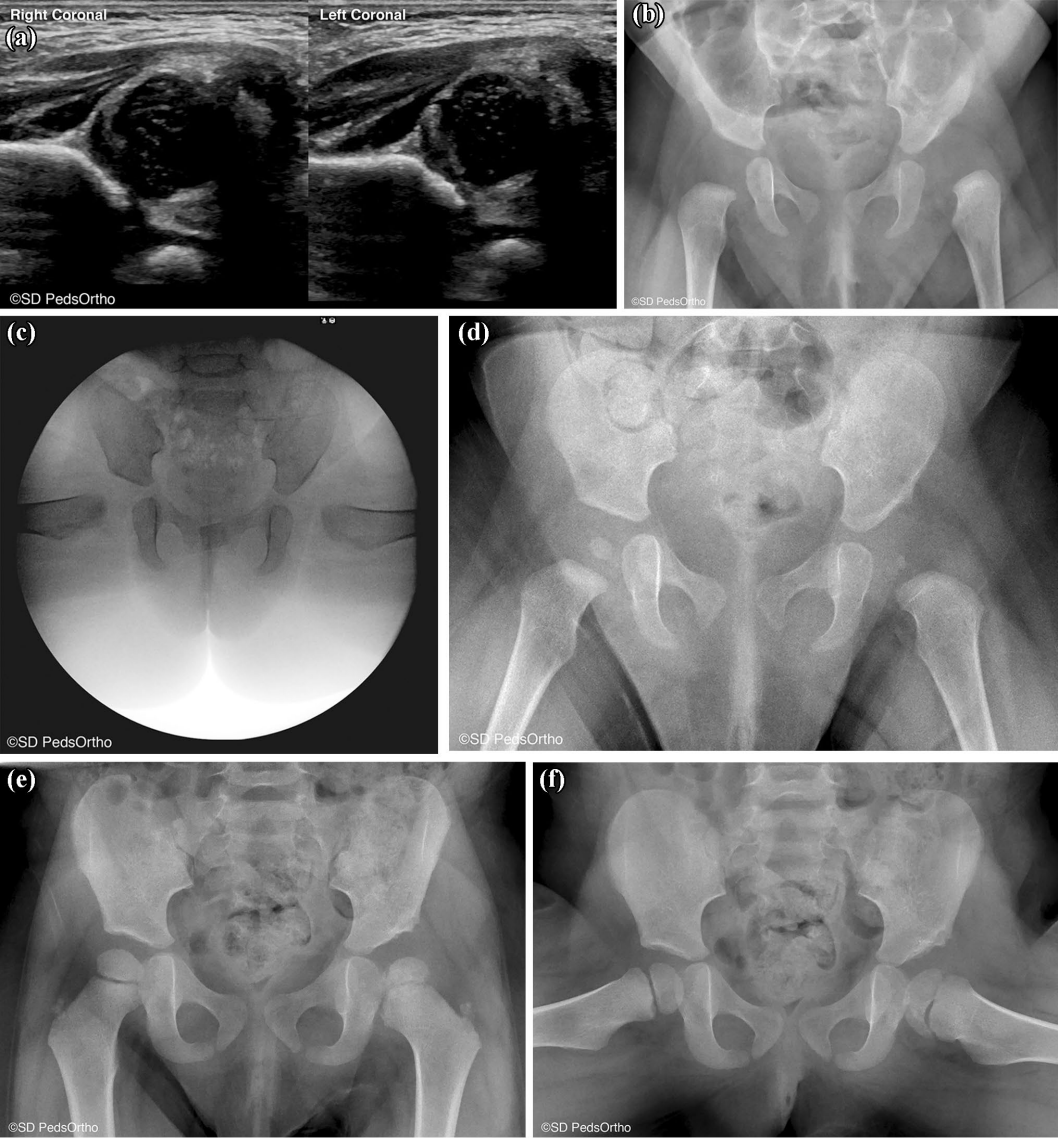
**A** Four-month-old first-born female with an Ortolani positive left hip, treated with a Pavlik harness. **B** After 3.3 weeks of Pavlik harness use, the hip remained IHDI III. **C** At 5.8 months of age an open reduction via medial approach was performed along with an adductor and psoas release. **D** At 6 weeks post open reduction, the hip remains reduced with significant dysplasia. **E** AP and **F** frog lateral view at age 2.5 years

The second large, multi-centered trial, is from Wada et al. from Japan [31]. They sent questionnaires to 12 institutes in Japan specializing in pediatric orthopedics in 1994 and 2008, evaluating the two groups of patients to look for changes in outcomes over time. Overall, they examined 4004 hips total (2481 in 1994 and 1523 in 2008). Of note, all of these were hips with frank dislocation. Reduction in the harness was possible nearly 80% of the time. The overall rate of ON was 13%. Of the proportion of hips followed to skeletal maturity, 74.6% were classified as Severin grade I or II (Fig. 2).

More recently, the International Hip Dysplasia Institute (IHDI) study group published on a smaller level 1 study evaluating brace treatment in infants with DDH. Upasani et al. included 204 hips in 159 infants that were treated with bracing, primarily using the Pavlik harness with a mean follow up period of 27 months [7]. The overall success rate of brace treatment was 79%. On multivariate analysis, variables associated with failure included femoral nerve palsy during treatment, treatment with a static brace, irreducibility (assessed to be Ortolani negative using ultrasound), age at treatment initiation > 7 weeks, right sided dislocation, and Graf IV hips.

### Failure of Harness Treatment

As mentioned above, the primary “complication” following harness treatment is treatment failure, or inability to reduce or maintain reduction of the hip. Mubarak et al. examined the reasons for harness failure in 18 patients [9]. They report that failures are typically secondary to poor application of the harness by orthopedists (most commonly inadequate flexion), with some attributed to poor parent compliance. They recommended weekly follow-ups following harness placement to evaluate for reduction, with abandonment of the harness if reduction was not achieved within 4 weeks. Typical failure rates for frankly dislocated hips are around 15–30% in the literature, and 0–2% for dysplastic and subluxated hips.

### Osteonecrosis Following Brace Treatment

Harris et al. published on 720 hips treated with the Pavlik followed for an average of 2 years [28]. They found that 11% were irreducible, and 9% had residual dysplasia at the end of harness treatment. They had a low rate of ON at 0.7%. They comment that the harness is not appropriate to use in children over the age of 8 months, or in whom 2–4 weeks of bracing does not reduce the hip.

Suzuki et al. published a series of papers examining the risk factors for ON with Pavlik harness use [21, 22]. In their largest series, they evaluated 270 hips with congenital dislocations treated with the Pavlik harness. Reduction was unsuccessful in 6%. Avascular necrosis was observed in 16% of the reduced hips, of which 27 hips were followed for an average of 9 years. 67% of these patients were classified as a Severin I or II at final follow up [22].

The age of patient at presentation is a well-recognized risk factor for Pavlik harness failure and osteonecrosis. Typically, studies report significantly better outcomes when treatment is initiated in patients under the age of 3 months. Some studies have examined results of Pavlik initiation in older children. Van de Sande et al. reported on 31 hips with late diagnosed hip dislocation treated with a Pavlik harness [8]. The average age at the start of treatment was 27 weeks, although it ranged from 21 to 57 weeks. They found an overall 65% success rate in reduction with the harness, with a 15% rate of ON. Only 2 patients (25%) with Tonnis grade 3 or 4 hips were able to be successfully reduced. Pollet et al. similarly studied 26 hips with late diagnosed DDH with an average follow up of 6.6 years [10]. The mean age at diagnosis was 9 months. Among this group, 46% were able to be reduced successfully after an average treatment of 14 weeks. Among the hips, they noted a 60% rate of reduction in Graf type 3 hips vs 0% in Graf type 4 hips, once again pointing to severity of disease being a critical factor in the success of the harness. Although the consensus is that the upper limit to attempt treatment with a Pavlik harness is 6 months, some studies have found worse outcomes with treatment beyond 4 months.

Interestingly, male sex as a risk factor for treatment was examined by one study. Borges et al. evaluated 78 congenitally dislocated hips in boys, noting that 93% of male patients treated with a Pavlik harness at a mean age of 7 weeks required additional methods of treatment [32].

### Femoral Nerve Palsy

Another known, albeit less common, complication of Pavlik treatment is the development of femoral nerve palsy. Murnaghan et al. evaluated all cases of femoral nerve palsy at their institution between 1992 and 2008, and found an incidence of 2.5% [20]. The vast majority of these (86.7%) presented at a week or earlier after initiation of treatment. In examining risk factors for femoral nerve palsy, these patients were on average older (56 vs 22 days), taller (55 cm vs 51 cm), heavier (4.8 vs 3.7 kg) with relatively elevated BMI (15.5 vs 14.3 kg/m^2^). Patients who developed a palsy were noted to have more severe dysplasia on presentation compared to the controls. Presence of a femoral nerve palsy was indicative of eventual failure of brace treatment, with only 46% of these patients undergoing successful treatment vs 94% of the controls. All patients with a femoral nerve palsy had return of function, although the time to recovery varied (average 5 days).

### Pavlik Harness Disease

Pavlik harness disease is a known complication with prolonged harness use. This is thought to be posterolateral remodeling of the acetabulum in response to prolonged flexion and abduction, worsening dysplasia and subsequent success of closed reduction. However, few studies in the literature document morphological changes with Pavlik harness disease with prolonged treatment of the irreducible hip. Gornitzky et al. performed a retrospective case series of 49 hips in 38 infants with DDH who failed Pavlik harness treatment [33]. Acetabular morphology was assessed with ultrasound using the change in alpha angle. They found no difference in alpha angle change in infants with Pavlik treatment for 3–5 weeks or those with prolonged wear.

## Complications with Open and Closed Reduction

The most complications after closed and open reduction include ON or PFGD, residual dysplasia, or re-dislocation requiring additional surgery. Overall, 33 papers were included [13, 34–64]. Osteonecrosis rates vary in the literature between 4 and 60%. Bradley et al. performed a meta-analysis specifically evaluating the rate of osteonecrosis following closed reduction for DDH. A total of seven papers were included with cumulative 538 hips. At mean follow up of 7.6 years, the overall rate of osteonecrosis was 10% following closed reduction [13].

We found only one study that evaluated a prospective, multi-center cohort looking at outcomes following closed reduction. Sankar et al. reported on 87 hips from several centers of the IHDI study group that underwent closed reduction at an average age of 8 months. At short term follow up of 22 months, they found an overall rate of osteonecrosis of 25%, with a 9% rate of treatment failure. 11% of hips required additional surgery within the short follow up period [34].

**Fig. 3:**
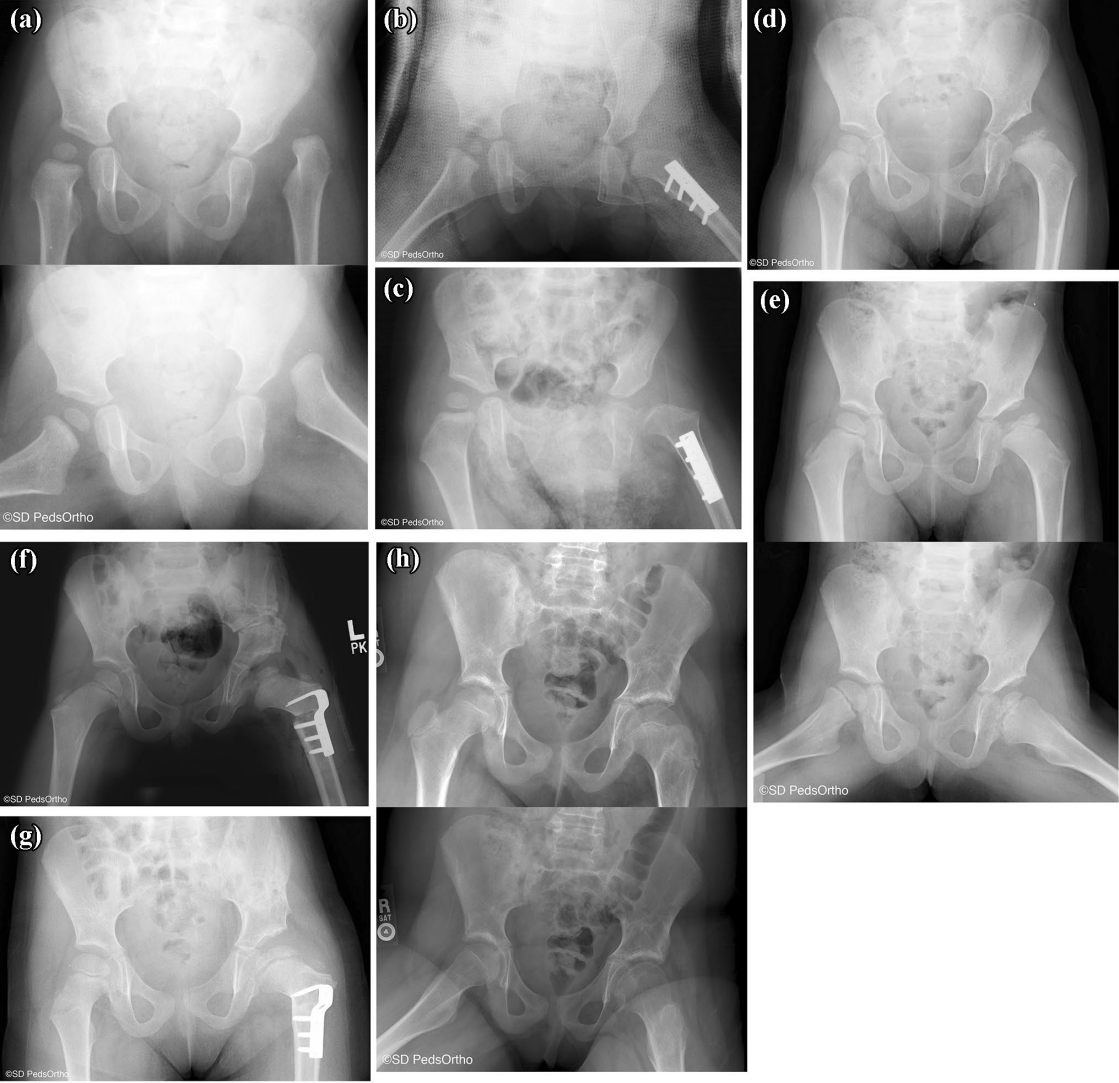
**A** AP and frog lateral view of a 13.8-month-old female with an IHDI IV left hip. **B** She was treated with open reduction and femoral shortening. **C** Three months post open reduction. **D** 1.4 years post open reduction, AVN is present. **E** 2.2 years post open reduction, residual dysplasia and femoral head deformity. **F** At 3.6 years of age she underwent a Salter osteotomy and varus derotational osteotomy. **G** 3.7 months post Salter procedure. **H** AP and frog lateral at 5.5 years post Salter osteotomy

Interestingly, Weinstein et al., evaluating closed reduction over a much longer follow up (152 hips over 31 years), noted that only 8.5% of hips required additional femoral or pelvic osteotomies following closed reduction [35]. They did note a 60% rate of osteonecrosis when using the Salter criteria of proximal femoral growth disturbance. 44% of hips developed a Kalamchi and McEwen grade II or higher osteonecrosis. 17 hips underwent total hip arthroplasty at an average age of 36, and 43% demonstrated degenerative changes at final follow up. The high rate of osteonecrosis in this paper is perhaps secondary to the older average age at reduction (21 months). They noted improved clinical outcomes and lower rate of growth disturbance when reduction was performed before 12 months of age. Similarly, Ponseti et al. reported on 8 to 29 year follow up after closed reduction in 40 dislocated hips in patients under the age of 12 months. They demonstrated that 92.5% of patients had good functional results, with a 20% rate of severe avascular necrosis [43] (Fig. 3).

### The Effect of Age

Older age at reduction has been implicated in several studies as a cause of worse outcomes. Zhang et al. specifically evaluated the impact of age following closed reduction [44]. They conducted a retrospective review of 107 patients with DDH, split into three groups based on age at reduction: < 12 months, 12–18 months and > 18 months. Logistic regression did not demonstrate an increased propensity for osteonecrosis among the older children. However, there was an increased risk of failure of closed reduction, residual dysplasia and future surgery in the older age groups. Other studies demonstrate similar results as well. In general, it appears that closed reduction does best when performed in patients under 12–18 months of age.

### Traction Prior to Reduction

**Fig. 4:**
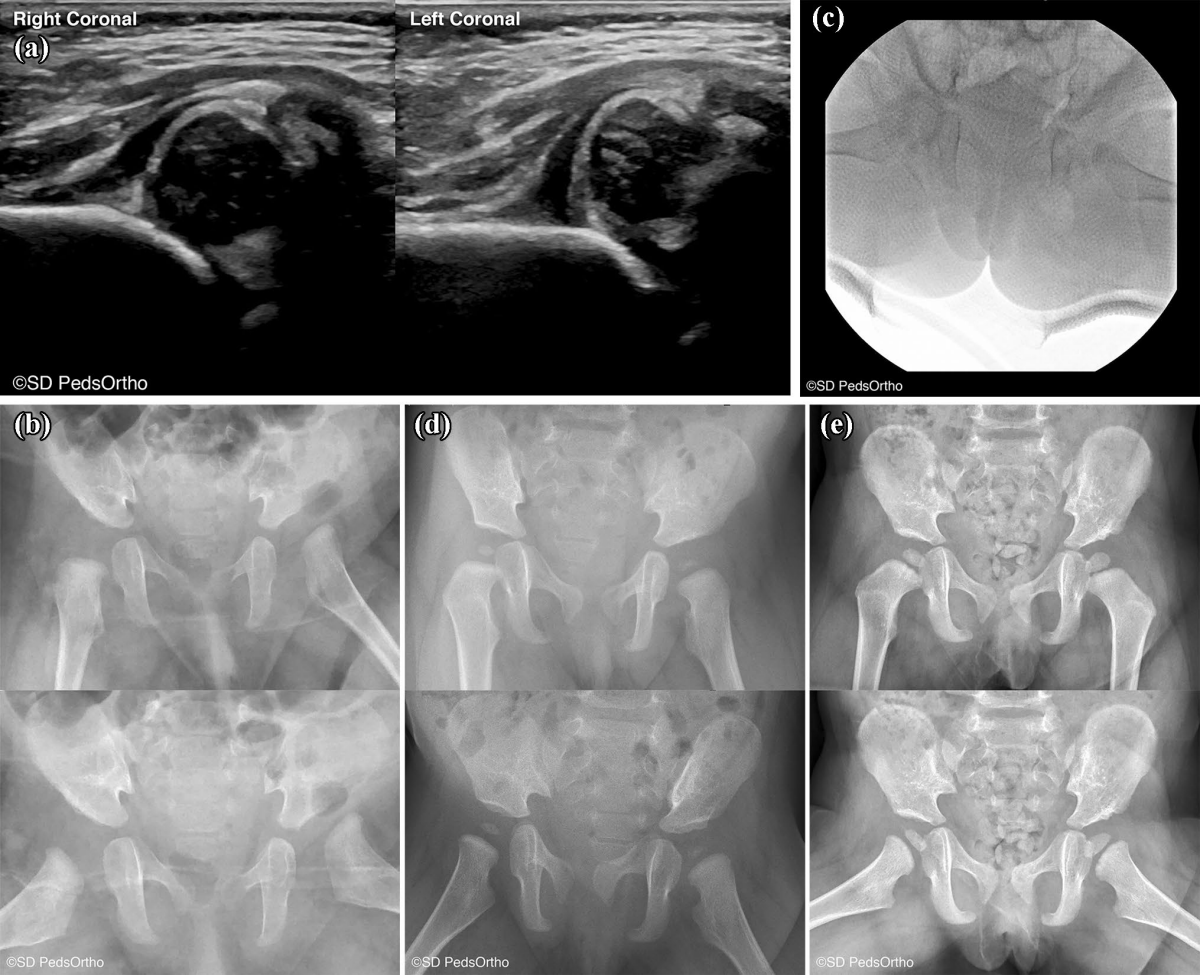
**A** A 1.6-month-old female with a Barlow positive right hip and dislocated and irreducible left hip treated with a Pavlik harness. **B** After 3.5 months of brace wear the left hip failed to reduce. **C** A closed reduction was attempted at age 4.9 months, this failed and was converted to an open reduction via medial approach with a adductor and psoas release as well as a capsulorrhaphy. **D** AP and frog 8.5 months post open reduction. **E** 20 months post open reduction

Controversy also exists about the use of traction prior to closed reduction. We found four studies evaluating the role of traction in closed reduction of the hip [48–51]. Park et al. performed a meta-analysis of studies specifically evaluating the role of pre-reduction traction on osteonecrosis, with a cumulative 683 hips included [48]. This found no significant difference in rates of osteonecrosis between groups. Other studies have found no difference in rates of success with closed reduction following traction as well. This has subsequently fallen out of favor, with most centers opting to perform an open reduction instead with concomitant osteotomy in cases with a high preoperative IHDI grade (Fig. 4).

### Open Reduction: The Effect of Approach Type

Traditional treatment in patients with a hip that is unable to be closed reduced, or presenting with a late dislocation, is open reduction. 43 papers evaluating open reduction were included in this review [14, 65–106]. A variety of approaches have been described. The Ludloff, Ferguson, and the Weinstein/Ponseti approaches are medial approaches through slightly differing intervals. The Ludloff medial open reduction has the longest track record and subsequently, the best studied. Pollet et al. reported on 13 year follow up in 58 hips after Ludloff medial open reduction [74]. They found a 19% rate of clinically significant AVN, and 22% of hips required additional surgery, of which 1 was secondary to re-dislocation. Overall, 78% of patients had a good or excellent result per Severin grading. Hips that underwent open reduction at an older age had worse outcomes (8.6 months vs 5.2 months). Konigsberg et al. reported on 40 hips at 10 year follow up with very similar results [80]. They noted a 15% rate of grade II or higher AVN, 20% rate of subsequent surgery and 1 re-dislocation. Again, age greater than 1 year was associated with higher rates of AVN.

Morcuende et al. reported on long term outcomes following the anteromedial approach described by Weinstein and Ponseti [70]. This approach is one interval anterior to the Ludloff approach, entering the joint between the neurovascular bundle and the pectineus. Although the reported rate of good to excellent outcomes by Severin grade was similar at 71%, they reported a 43% rate of ON. Some of this is possibly secondary to an average age at reduction of 14 months. 26% of patients had residual dysplasia and 17% required additional surgery.

**Fig. 5:**
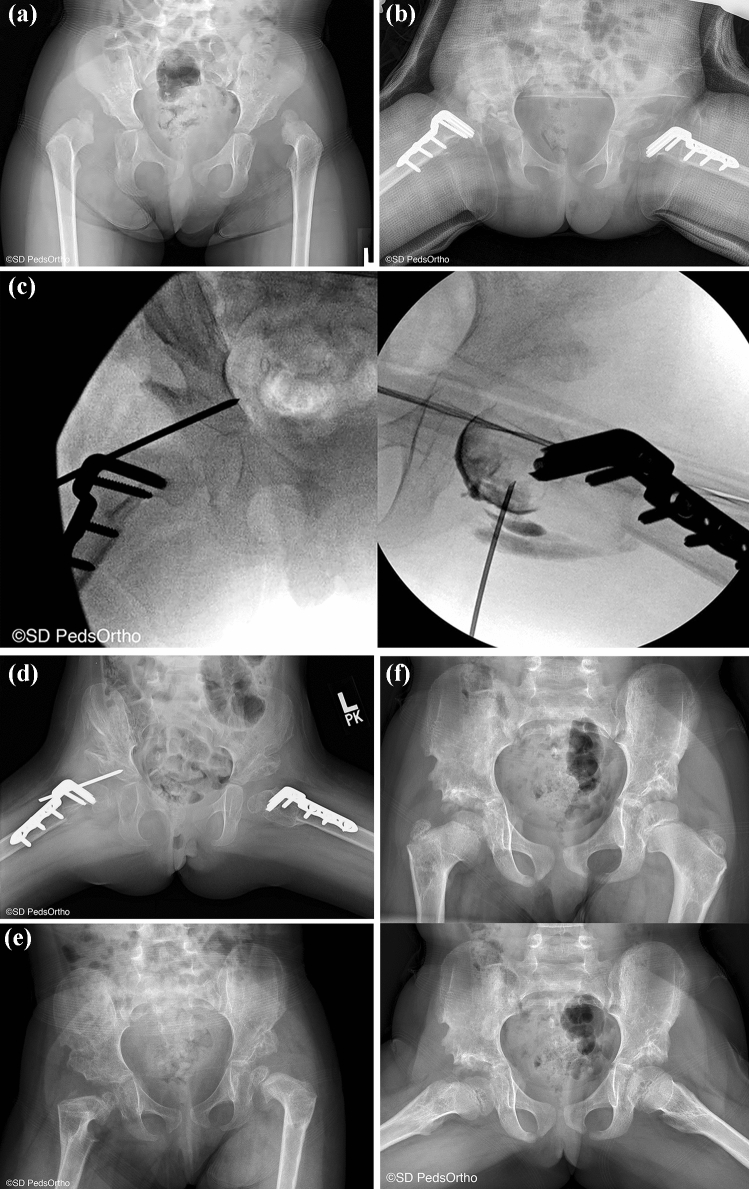
**A** A 3-year-old female with bilateral hip dislocations. **B** She was treated with bilateral open reduction and femoral shortening procedures. **C** The right hip was found to be dislocated posteriorly and was taken back to the operating room and a repeat open reduction was performed, along with a capsulorrhaphy and the hip we held in place with a k-wire. **D** Six weeks post k-wire fixation. **E** Six months post k-wire fixation. **F** AP and frog lateral X-ray 3 years post k-wire fixation

We found two papers that reported on outcomes following the Ferguson approach, i.e. medial to the Ludloff interval between the adductor longus and the graciles [79, 81]. Interestingly, reported outcomes following this approach appear to be significantly better that the other two. Tumer et al. studied 57 hips that underwent a Ferguson approach at an average age of 11 months, with average follow up of 8.1 years [81]. 98% of hips were categorized as a Severin I or II, and the rate of AVN was only 8.9%. The rate of secondary procedures remained the same at 19%. Similar results were found by Kiely et al., with 92% of hips rated Severin I or II, and a 6% rate of AVN [79] (Fig. 5).

Studies evaluating isolated anterior open reduction are rare, since this approach is typically chosen in an older child in concert with pelvic osteotomies. We found one paper directly comparing the anterior approach to the medial approach [75]. With 21 hips in the medial approach group and 22 in the anterior approach group, no significant differences were found with respect to clinical or radiographic outcomes, rate of AVN, or need for subsequent surgery. Novais et al. conducted a meta-analysis to evaluate the association between occurrence of osteonecrosis and medial vs anterior open approaches, including 9 studies reporting on the medial approach in 364 hips, and 8 studies reporting on the anterior approach in 220 hips [14]. After controlling for age, they found no difference in rates of AVN (18.7% for medial vs 19.6% for anterior). Contrary to other studies previously outlined that report worse outcomes with increasing age for both closed and open reduction, they found no difference when looking at reduction before or after 12 months of age. The rate of osteonecrosis with closed reduction was 8.0% before a year of age and 8.4% after. This was lower than reported rates for open reduction, which was 18.3% before a year of age vs 20.0% after.

### Early vs Late Reduction?

Some controversy exists regarding very early (prior to presence of the ossific nucleus) reduction as well. Some authors have postulated that delaying reduction until after the presence of an ossific nucleus may be protective against the development of osteonecrosis [56–64]. Segal et al. evaluated the rate of AVN in the presence or absence of the ossific nucleus, either on ultrasound or radiographs [62]. They found that only 4% of hips with an ossific nucleus developed osteonecrosis in their study, compared to 53% of the hips where the ossific nucleus had not developed yet. However, this finding has not been borne out by three subsequent meta-analyses. The most recent of these, by Chen et al., included 21 studies and found that osteonecrosis developed in 20.4% of hips with an ossific nucleus compared with 21.2% of hips where the ossific nucleus was absent [107].

### Combined Open Reduction and Pelvic Osteotomy

There is also some controversy regarding the treatment of neglected DDH in patients over 18 months of age. This has increasingly transitioned to single stage open reduction and pelvic and/or femoral shortening osteotomies (ORPO) rather than just open reduction, assuming that the acetabulum would not remodel on its own past this point. The largest study evaluating ORPO is by Ning et al. [93]. They report on a retrospective review of 864 hips with 6 year follow up. 85% of hips had a good or excellent Severin grade, and 79% had a good or excellent McKay score. AVN was noted in 27.4% of hips, 1.6% had a re-dislocation and 3% had residual dysplasia requiring additional surgery.

Wedge et al. reported on 45 year outcomes in Salter’s originally described cohort [96]. This included 101 hips that underwent preoperative traction, open reduction, capsulorrhaphy, and innominate osteotomy between 1958 and 1965. At 45 years, hips that underwent this procedure had a 54% survival rate. This was a sharp decline compared to a 99% survival at 30 years and 86% at 40 years post operatively. A subset of this group of patients with long term follow up (78 hips) was compared with long term outcomes of dislocated hips treated with closed reduction (58 hips) alone. At 48 years of follow up, 50% of hips that underwent closed reduction survived vs 69% of hips that underwent ORPO. 17% of hips in the closed reduction group and 22% of hips in the ORPO group required additional surgery for subluxation, dislocation or residual dysplasia.

### Limitations

This narrative review reports on common complications following treatment of DDH in children less than four years of age. This study has some limitations. A limitation common to narrative reviews is that PRISMA guidelines were not adhered to as one might with a systematic review. Given the breadth and scope of the paper, it was difficult to precisely define inclusion criteria for articles, resulting in a varying range of methodology in the selected papers. This made it impossible to meaningfully pool data to determine the relative prevalence of different complications. We found very few papers in the literature that specifically sought to identify all complications following differing DDH treatments in a cohort. As a consequence, one of the sources of bias in this study is that common or more serious complications such as ON are over-represented in our search, with fewer papers specifically addressing less severe outcomes such as residual dysplasia. In addition, our attempt to highlight longer term studies or studies with larger study populations resulted in a preponderance of papers from certain centers such as the University of Iowa, or international study groups such as IHDI. Despite these limitations, our review of the literature demonstrates a few key highlights.

## Conclusion

Although there is excellent potential for a good outcome when DDH is diagnosed and treated prior to four years of age, osteonecrosis continues to be a concern with all treatment methods. A subset of patients from this young cohort will continue to have residual dysplasia or recurrent dislocation requiring return to the operating room. In the long term, hips without complications related to DDH treatment tend to do well, although a significant percentage of them will inevitably require joint replacement surgery.
